# Does COVID-19 lockdowns have impacted on global dengue burden? A special focus to India

**DOI:** 10.1186/s12889-022-13720-w

**Published:** 2022-07-22

**Authors:** Hemlata Sharma, Ashal Ilyas, Abhiroop Chowdhury, Nitesh Kumar Poddar, Anis Ahmad Chaudhary, Sireen Abdul Rahim Shilbayeh, Alnada Abdalla Ibrahim, Shahanavaj Khan

**Affiliations:** 1 Department of Biosciences, Manipal University Jaipur, Dehmi Kalan, Jaipur-Ajmer Expressway, Jaipur, Rajasthan 303007 India; 2 Department of Biotechnology, Invertis University, Bareilly, 243123 India; 3 Jindal School of Environment and Sustainability, O.P. Jindal Global University, Sonipat, 131 001 Haryana India; 4 Department of Biology, College of Science, Imam Mohammad Ibn Saud Islamic University, Riyadh, Saudi Arabia; 5 Department of Pharmacy Practice, College of Pharmacy, Princess Nourah bint Abdulrahman University, P.O. Box 84428, Riyadh, 11671 Saudi Arabia; 6 Department of Health Sciences, Novel Global Community Educational Foundation, Hebersham, NSW Australia; 7 Department of Medical Lab Technology, Indian Institute of Health and Technology (IIHT), Deoband, 247554 Saharanpur, UP India; 8 Department of Pharmaceutics, College of Pharmacy, King Saud University, PO Box 2457, Riyadh, 11451 Saudi Arabia

**Keywords:** COVID-19, Dengue, Human behavior, Cross-reactivity, Lockdown

## Abstract

**Background:**

The world has been battling several vector-borne diseases since time immemorial. Socio-economic marginality, precipitation variations and human behavioral attributes play a major role in the proliferation of these diseases. Lockdown and social distancing have affected social behavioral aspects of human life and somehow impact on the spread of vector borne diseases. This article sheds light into the relationship between COVID-19 lockdown and global dengue burden with special focus on India. It also focuses on the interconnection of the COVID-19 pandemic (waves 1 and 2) and the alteration of human behavioral patterns in dengue cases.

**Methods:**

We performed a systematic search using various resources from different platforms and websites, such as Medline; Pubmed; PAHO; WHO; CDC; ECDC; Epidemiology Unit Ministry of Health (Sri Lanka Government); NASA; NVBDCP from 2015 until 2021. We have included many factors, such as different geographical conditions (tropical climate, semitropic and arid conditions); GDP rate (developed nations, developing nations, and underdeveloped nations). We also categorized our data in order to conform to COVID-19 duration from 2019 to 2021. Data was extracted for the complete duration of 10 years (2012 to 2021) from various countries with different geographical region (arid region, semitropic/semiarid region and tropical region).

**Results:**

There was a noticeable reduction in dengue cases in underdeveloped (70–85%), developing (50–90%), and developed nations (75%) in the years 2019 and 2021. The dengue cases drastically reduced by 55–65% with the advent of COVID-19 s wave in the year 2021 across the globe.

**Conclusions:**

At present, we can conclude that COVID-19 and dengue show an inverse relationship. These preliminary, data-based observations should guide clinical practice until more data are made public and basis for further medical research.

## Highlights

• COVID-19 has increased the burden on the health care system across the globe.

• COVID-19 has inverse relation with the occurrence of Dengue cases.

• Dengue situation is worse in countries with low GDP.

• Human behavior and social distancing have direct correlation with the number of Dengue cases.

• Cross-reactivity or overlap between Dengue and COVID-19, has proportional effect on each other.

## Background

COVID-19, the current pandemic affecting millions of people globally, is one of the major health emergencies in the world. COVID-19 originated from Wuhan, China, in December 2019. Gradually, the whole world was affected by the disease [1]. It showed symptoms ranging from mild respiratory issues to acute pneumonia. COVID-19 is an infectious disease that spreads through human contact due to SARS-CoV-2 [2]. This infectious disease spreads not just from the droplets produced by sneezing or cough of a COVID-19 infected person; but even simple exhalation and speaking can also transmit. The main symptoms of COVID-19 are high fever, cough, and respiratory problems [1, 3, 4]. SARS-CoV-2 is the third member of the COVID-19 virus family to produce respiratory disease in humans after SARS-CoV and MERS-CoV [2].

The primary source of COVID-19 was observed via bat species which was sold as live food at the seafood market in Wuhan city [5]. Presently, every country is primarily focused on to reduce R_0_ below 1 by public health and social measures and through high vaccination coverage rates to combat COVID-19 disease throughout the world. Since there is a rapid increase in confirmed cases and deaths by COVID-19 with limited available medicinal therapy, the only protective measure is to restrict human social indulgence by imposing lockdowns. Almost all COVID-19 affected nations-imposed lockdown and home quarantine to regulate human social behavior in their surroundings. Due to imposed restrictions, human social behavior is greatly regulated as people are not allowed to travel frequently to other countries or even to a different city or state which made them remain isolated in their home.

The current pandemic has affected the health system, especially in the developing countries in an unprecedented crisis. Around 14.3% of COVID-19 patients have been found to be affected by secondary infections such as tuberculosis (TB), influenza chronic hepatitis, and other concurrent infections [6]. In India, it has been estimated that TB affected around 2.64 million people in the year 2019, which further led to a mortality rate of 1000 per day [7]. It has been observed that the number of TB cases in India was the highest in the world during the pandemic time [8]. TB has majorly affected immunocompromised patients with HIV, diabetes mellitus and SARS-CoV-2 [9]. In the case of TB access to adequate nutrition is also a key consideration, but during a pandemic lockdown, the economic difficulties may have impacted the ‘access to a healthy diet’, due to the paucity of employment opportunities for the socio-economically marginalized section of Indian society. Cramming in crowded spaces due to lockdown may also have impacted the spread of TB infection during the lockdown.

On the other hand, co-infections of opportunistic pathogens increase tremendously during the pandemic of COVID-19. During the second wave of COVID-19 in India, an outbreak of mucormycosis, which is affecting thousands of COVID-19 patients and thus aggravating the conditions of an epidemic which leads to the fatality rate of 50% [10, 11]. There was an urgent need for adequate countrywide surveillance, diagnostic, and robust health management system, with respect to public awareness to alleviate the burden of co-infection of fungus and COVID-19 [12–14]. In context to the zoonotic virus of COVID-19, in the Republic of Guinea, a Marburg virus disease (MVD) outbreak was observed during the pandemic of COVID-19 with a fatality rate of 90% [15]. In addition to that, outbreaks and spread of cholera in Africa and other infections such as hepatitis in Egypt have been reported in recent years [16, 17]. All these challenges reflect the burden on the health system with preventive measures like hygiene, sanitation and proper diagnostic tools to control the outbreak of infectious diseases especially in the African countries [18]. Nations’ health resources were focused to manage the cases of COVID-19 which may have resulted in neglecting treatment-management of other diseases favouring their spread, as well as increased infection rates during the pandemic period. COVID-19 significantly weakens the immunity of the patient making them more prone to contract other diseases.

Several diseases are common in the tropical climate and act as non-communicable diseases in low- and middle-income tropical-subtropical countries. Underdeveloped health care system and poor sanitation are the major hurdles to managing these diseases. Similarly, spread of vector borne-diseases such as dengue are usually related to these factors such as human behavior, sanitation system, mosquito breeding sites and rapid changes in urban infrastructures, increasing population density, rural to urban migration patterns and the people’s exposure while traveling to different places with varied climatic conditions [19].

According to WHO, about half of the entire human population lives in the vector-borne disease-prone areas, resulting in approximately 1 million death each year [20]. Among several other vector-borne diseases, dengue is the fastest spreading mosquito-borne disease caused by *Aedes* mosquito. The virus spreading dengue belongs to the *Flaviviridae* family and contains positive-strand RNA as genetic material [21–24]. Due to the increased incidence of disease among the human population, global health concerns were raised across the world. Apparently, with the emergence of this disease as an endemic, many countries were investigating methods of its prevention even before the COVID-19 outbreak. COVID-19- related changes in health policy and priorities have affected the dengue control and management program [25]. The situation of COVID-19 resulted into lockdown around the world for 3–4 months, which affected the door-to-door dengue survey and dengue site control activities [26, 27]. It can affect mortality in dengue cases. Dengue fever usually has a mortality rate of less than 1%, which increases to 2–5% in the case of severity. In comparison, COVID-19 mortality largely depends on many factors, such as age, immunity, and other morbidities [28, 29]. Dengue fever and COVID-19 are unique cases to differentiate because they share clinical and laboratory characteristics [30]. Some researchers mentioned cases that were wrongly diagnosed as dengue but later confirmed to be COVID-19 [31]. In addition, co-infections with arboviruses and SARS-CoV-2 have not been well studied.

Few studies have shown the relationship between the impact of COVID-19 Lockdowns and social behaviour on the number of Dengue cases in different parts of the world including southeast Asian countries [32–34]. But the detailed and systematic analysis of the effect of COVID-19 lockdowns with respect to the social behavior of people on the number of dengue cases in different parts of the world during the pre-pandemic and pandemic of COVID-19 is still not well studied.

Considering the changing indoor and outdoor activities and environment under the influence of lockdown, social distancing during the COVID-19 outbreak, the impact on dengue cases could be analyzed and marked for future investigation [35]. This lockdown provided the perfect experimental set up to understand the role of behavioral changes, lockdown, and closures of public spaces on proliferation of the disease. The present research aims to find the connection between COVID-19 lockdown and global dengue burden.

This article is focused on this major connection between the two diseases plaguing the modern era to give insight into major public health lessons learned during this present pandemic.

## Methods

**Fig. 1:**
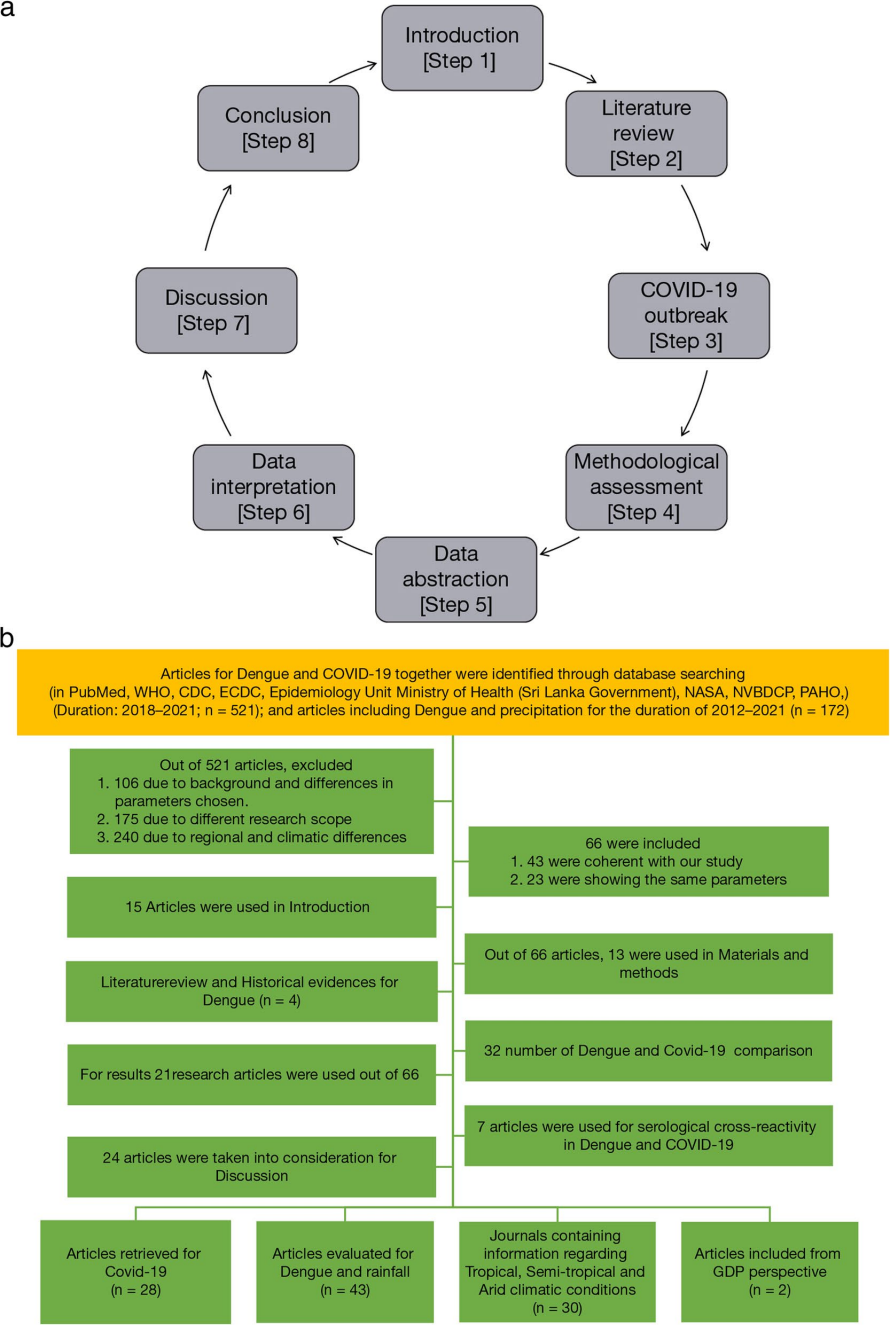
**a** This flow-chart provides the specific steps in relation to meta-analysis process **b** Selection methodology of research articles with different climatic parameters (arid, semi-arid and tropical) and GDP as per the PRISMA format

In this article, we have screened secondary data and available literature from 2012–2021, to understand the effect of rainfall on dengue cases and access the correlation between dengue cases and COVID-19 during 1^st^ and 2^nd^ waves of pandemic [36, 37]. We used different websites, such as PubMed, WHO, CDC, ECDC, Epidemiology Unit Ministry of Health (Sri Lanka Government), NASA, NVBDCP, and PAHO. We took a range of countries while keeping different parameters in consideration. Countries in arid region (e.g., Australia, Cambodia, Brazil); semiarid region (semiarid states of India and Pakistan); and tropical region (Honduras, Nicaragua, Colombia, Malaysia, Philippines, and Vietnam) are included. Same conditions were used with Indian states as well, namely arid region (Rajasthan and Gujarat); semiarid zones (Maharashtra, Bangalore, Tamil Nadu, Andhra Pradesh and Madhya Pradesh); and tropical zone (Telangana, Kerala, Orissa, and West Bengal) are included. Another framework, which was used to collect the data, is the economic polarity (marginalized or affluent). Countries such as Nicaragua, Pakistan, and Cambodia are underdeveloped. India, Malaysia, Philippines, Colombia, Vietnam, Cuba, Sri Lanka, Panama, and Nepal are developing nations, whereas the United States and Australia are included in the developed category. In this article, our focus was on different classifications and their significance with dengue cases and COVID-19 pandemic. In this context, another important feature was the cross-reactivity between these two viruses and their mode of action inside a host cell. Figure 1a demonstrates the specific steps in relation to meta-analysis process.

### Patient and public involvement statement

This research has been conducted using secondary data from available literature and open sources. There is no involvement of hospitals or hospital-centric data. We are using only secondary data for our research articles. So, ethical approval is not needed in this case.

This article includes eight major categorizations as mentioned in Fig. 1a, of which the major steps are data abstraction and interpretation with the help of which the correlation between dengue and COVID-19 might be substantiated.

The data on dengue cases were collected from Rajasthan, Gujarat, Andhra Pradesh, Bihar, Karnataka, Maharashtra, Punjab, Rajasthan, Uttar Pradesh, Uttarakhand, and Telangana to explain the seasonal trends of dengue in arid, semiarid, and tropical regions of India [38].

In order to understand the seasonal trends of dengue and COVID-19, we obtained the data of COVID-19 cases from WHO data sources. We included data from January 2020 to the last week of June 2021. In this data, cases of COVID-19 recorded around the globe have been studied. The process followed the PRISMA (Preferred Reporting Items for Systematic Reviews and Meta-Analyses) protocol (Fig. 1b).

Apart from these, 5 years of research articles on vector-borne diseases, behavioral changes due to the COVID-19 pandemic were studied to understand the human behavioral effect on these diseases.

### Dengue and its impact on global health

At present, dengue fever is found in about 129 countries in the world. Every year, 390 million people in the world are at risk of falling ill due to dengue virus [39, 40]. The first dengue case was seen in 1750 in Asia, Africa, and North America [41, 42]. In contrast, the first case of DHF (dengue hemorrhagic fever) was reported in 1950 in Southeast Asia [43–45]. According to the WHO, 2.38 million cases of dengue were reported in the United States during 2016. About 1.5 million cases were reported in Brazil alone. The first case of dengue was reported in Afghanistan in 2019. In 2019, the Philippines and Vietnam had the highest number of dengue cases in Asia. The rapid rise in the number of confirmed cases obeys a variety of influential factors discussed and described later in this paper.

### Historical records on the spread of dengue in India

When analyzing the data related to dengue cases, it was found that the first outbreak of dengue in India was observed in Calcutta in 1963 [46, 47]. After this, an outbreak of dengue was recorded in Madras in 1989–90 [48]. The dengue epidemic and its spread patterns had changed in India after 1990. It gradually spread to almost every part of India, infecting thousands in the states of Andhra Pradesh, Gujarat, Chandigarh, and Goa that were previously not affected by the disease. *Aedes* sp mosquitoes’ population started increasing and their metapopulation migrated to remote corners of the nation, which led to the spread of disease in urban and rural areas [49]. Over the last decade, increased population growth, a rise in unemployment, and decrease in profit from agriculture, increase in agricultural debt-related suicides, particularly in the cotton cultivation belts of central India- the western Vidarbha region, have instigated rapid urbanization and resulted in rural–urban migration of population. This had led to a rise in the quantity of domestic waste, crowded urban suburbs, a rise in urban slums and improper management of rainwater during monsoon months, which in turn increased the spread of dengue vector by increasing the breeding sites. India witnessed a rapid increase in dengue cases during the past years, requiring immediate attention, scientific investigation, and an everlasting cure.

### The COVID-19 outbreak

The first case of COVID-19 was registered in December 2019 in Wuhan, China, which began to spread worldwide since January 2020 [50]. According to the WHO, the total numbers of worldwide COVID-19 cases were 180,858,520 as of June (2021) [51]. Out of total cases, the total number of fatalities were counted as 3,918,130 during the mentioned period.

The causative agent of COVID-19 mutation got serious when it was mutated, (Scientific name: *B.1.617*) due to which it became more deleterious and transmissible in humans. The second wave of COVID-19 proved to be more disastrous and fatal in comparison to the first one which was more transmissible in its nature. As per data, across the globe, around more than 3.9 million lives have been lost as of now and still, the second wave has not yet left completely. In India itself, 393,338 deaths took place during the first and second wave of COVID-19.

### COVID-19 outbreak in India

The first case of COVID-19 in India was registered on 30 January 2020 in Kerala [52]. According to the WHO report, as of 9 March 2020, COVID-19 cases in India reached from 3 to 44. During March, more cases of COVID-19 were reported in the states of Kerala, Uttar Pradesh, Haryana, and Karnataka. After March, Rajasthan, Madhya Pradesh, Gujarat, and Tamil Nadu witnessed a rise in COVID-19 cases. Additionally, Maharashtra and Delhi, too, also witnessed a rapid hike in the number of infected persons.

### Impact of the second wave of COVID-19 in India

COVID-19 has a serious impact on India’s health infrastructure. Between 2020 and 2021, there was a reduction in reported cases making the population as well as policy makers complacent about their capability to fight the virus. Lockdown rules were relaxed, and people started neglecting the cautions associated with COVID in that period. Hence, when the second wave hit India, the situation turned dire, almost rapidly. At a point in time during the second wave reported cases have taken a massive leap to 30,134,445 amongst which 393,338 deaths were reported and 29, 128, 267 cases recovered [51, 52].

The situation got worse due to the rapid mutability of the virus resulting in more cases, morbidity, detection of new co-morbidities, and dearth of essential life services and increased mortality, particularly in children. This has resulted in a massive health burden on the nation and stretched its public health infrastructure to its limit which indirectly has also affected the provision of these vital services. SARS-CoV-2 mutated rapidly, and double mutant strains were also present and located from various spots of India [53, 54]. This indicates the evolution of the virus in response to changing conditions, increasing its transmissibility, and pathogenicity. In the first wave only, older patients got infected with the disease and people having co-morbidities have shown a spiked mortality rate. But in the same period, younger populations were relatively safe. But the situation was altered during the 2nd wave when patients in the age group of 25–50 years also showed increased mortality [55].

### Comparative analysis between dengue and COVID-19 virus life cycle

**Fig. 2:**
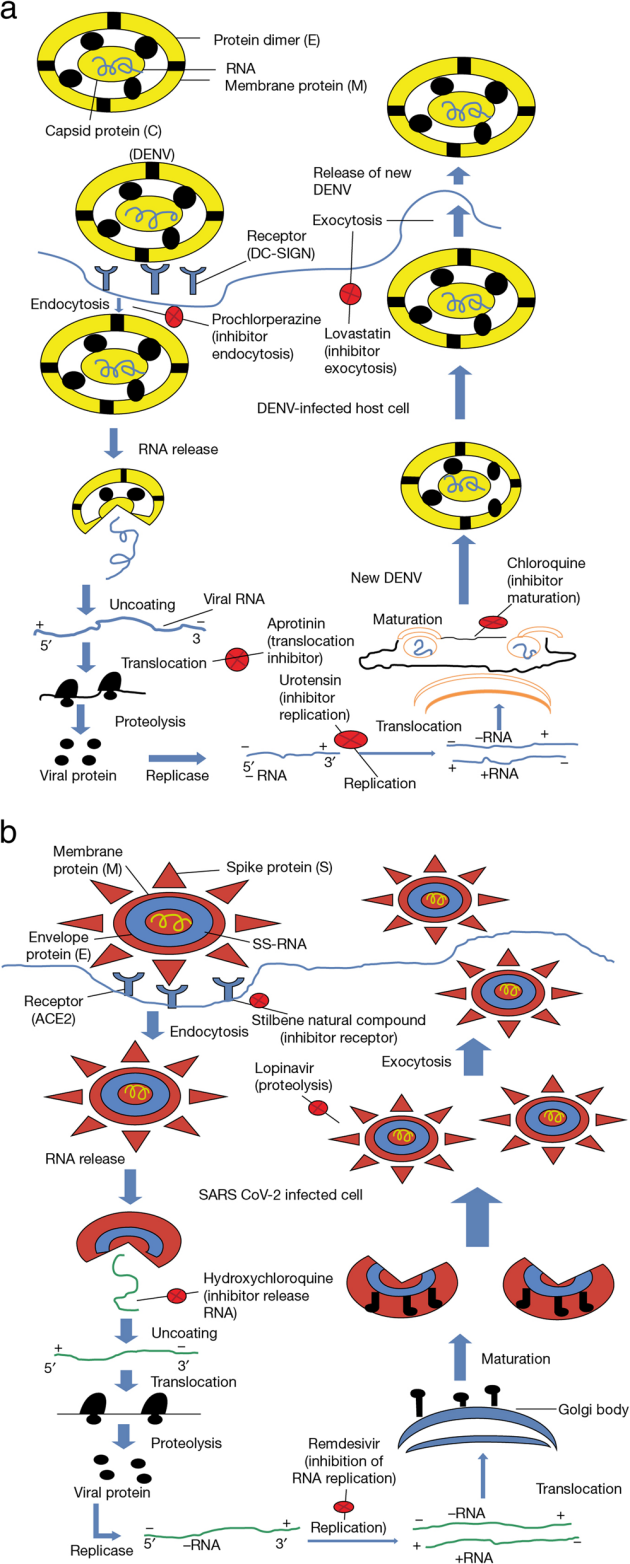
**(a-b)** Comparison between life cycles of vector-borne virus and COVID-19 virus. **a** Stages of DENV (dengue virus) life cycle, DENV virus with capsid protein and RNA bind to host cell receptor (DC-SIGN). Dengue viral proteins are coded from one mRNA. Inside the infected host cell, DENV releases RNA which simultaneously translocates, leading to proteolysis of viral protein. Replicase synthesizes RNA which goes for maturation inside a new viral DENV particle, which releases outside of the host cell through exocytosis. Figure 2a also represents some inhibitors intervening in the replication process of viral particles inside a host cell. **b** The SARS-CoV-2 contains spike protein, membrane protein, envelope protein and single-stranded (SS-RNA). Spike proteins (S) binds to the host surface receptors (ACE2). SARS-CoV-2 viral proteins are coded from subgenomic mRNA. The virus internalized after attachment through endocytosis. In the next stage, SARS-CoV-2 releases its RNA into the host cell turning it into SARS-CoV-2 infected cells. The released RNA undergoes uncoating followed by translocation and proteolysis of viral proteins. Replicase enzyme synthesizes new RNA which after maturation develops into new viral particles and releases outside the cell through exocytosis. Figure 2b also represents some inhibitors, for example, hydroxychloroquinone, lopinavir intervening the replication process of viral particles inside the host cell

Table 1 shows that both COVID-19 [1] and dengue virus has positive SS-RNA and three nonstructural proteins. Besides this, both viruses exhibit a similar replication process after entering the cell (Fig. 2) and they differ at a few stages during replication processes: Dengue viral proteins are coded from one mRNA, and in contrast, several SARS-CoV-2 viral proteins from subgenomic mRNA.

**Table 1:** Comparison between SARS-CoV-2 and Dengue virus [1, 56, 57]

Features	Dengue	COVID-19
Cause	DENV	SARS-CoV-2
Emergence date	1780	December 2019
Place of emergence	Philadelphia	Wuhan, China
Entry receptor	Heparan sulphate/DC-SIGN	ACE2
Genome type	Positive SS-RNA	Positive SS-RNA
Protein composition	Three structural and seven non-structural protein	16 proteins from ORF1a and 1b, and three structural protein, N protein and the other accessary proteins from subgenomic mRNA
Genomic size	11 kb	27–32 kb
Symptoms	High fever, rashes on-skin, nausea, vomiting, joint pain	Fever, cough, breathing difficulties, sneezing, vomiting
Diagnostic method	RT-PCR, ELISA, NS1 antigen test	RT-PCR, ELISA, CT-Scan
Total infected countries	129	227
^#^Total cases	390 million/year	149 million/year
^#^Total deaths	Estimated 40,000/year	2,009,163/year
Endemic/Pandemic	Endemic	Pandemic
Cured/Or Not Cured	Not yet	Not yet

^*^DC-SIGN Dendritic cell-specific intercellular adhesion moleculer-3-grabbing non-integrin

^*^ACE2 Angiotensin converting enzyme 2

^*^SS-RNA Single-stranded RNA

^#^Source: https://www.worldometers.info/coronavirus/?Si

SARS-CoV-2 is included in the family COVID-19 viridae [58]. In addition, Table 1 also describes that COVID-19 virus RNA genome size is between 27 and 32 kb [1, 56, 57]. It consists of three main structural proteins, spike (S), envelope (E), membrane (M), and 16 nonstructural proteins [1, 56, 57, 59, 60] (Table 1). Attachment and internalization of the COVID-19 virus into the host is mediated by the presence of spikes on the virus surface (Fig. 2b). The protein for replication of the virus, formation of spike, and nucleo-capsid are encoded by the gene present on ORF1 downstream region of COVID-19 virus [61].

On the other hand, the structure of the dengue virus includes three structural proteins, such as capsid (C), membrane (M), and envelope (E). In addition to this, seven nonstructural proteins (NS1, NS2A, NS2B, NS3, NS4A, NS4B, and NS5) are also found in it [21–23, 41]. The RNA genome size of dengue is 11 kb [22, 41]. Dengue virus enters the cells through clathrin-mediated endocytosis [60]. Studies suggest that the virus interacts with different receptors on the surface of the cell and moves toward the existing clathrin-coated vesicles [61] (Table 1). When comparing the lifecycle of both the viruses (Fig. 2a and b), it is found that in the first phase of the COVID-19 virus life cycle, spike protein binds to the host cell ACE2 (angiotensin-converting enzyme 2) receptor [4, 56, 59, 60, 62–65]. The first phase of the dengue virus life cycle binds to the heparan sulphate/DC-SIGN (dendritic cell-specific intercellular adhesion molecule-3-grabbing non-integrin) receptor present on the host cell with the help of protein E [22, 41] (Table 1). The different types of life cycles utilized by both the viruses for infection might be one of the factors that relate dengue with COVID-19 through positive or negative correlation. The viral life cycle decides the entry of the virus into the host and its replication under different environmental and physical conditions [66].

DENV surface proteins and the components of the plasma membrane are majorly responsible for the process of recognition between the DENV and the targeted cell (Fig. 2a). Usually, the binding is nonspecific in nature, which in turn leads to the accumulation of virus moieties onto the cell [67]. Although several attempts have been made in order to describe the molecule and its nature is responsible for DENV to interact with the cell, but any particular specific candidate has not been described so far but still several molecules have emerged into the picture for instance mannose receptor (MR) and adhesion molecule of dendritic cells (DC-SIGN).

In order to contain this pandemic, information on the infection mechanism of SARS CoV 2 and its role in destabilizing the host’s cellular metabolism need to be focused on. The virus uses the ACE2 receptor in addition to human proteases to get entry inside the host’s cell (Fig. 2b). Once it enters, it can evade the immune system which further leads to infection at the cellular level and the virus spreads across the whole body [68].

## Results

### Variable relationship between rainfall and dengue cases before COVID-19 scenario

#### Seasonal trend of dengue across the globe

Climatic conditions play an essential role in spreading and increasing dengue fever. Above-average rainfall, high humidity, and daily temperature of 25 °C to 30 °C are suitable for the survival and breeding of dengue vectors [69]. In these ideal conditions, the chances of dengue outbreak are increased due to increased breeding.

For this study, different countries were selected based on demographic, weather, and socioeconomic criteria. In this selection, gross domestic product (GDP), dengue cases, and geographical location of those countries were considered as the basis. This article focused on the pattern of dengue cases under different geographical conditions and the ubiquity of dengue conditions in low-income countries (Tables 2, 3, and 4). Dengue cases are more prevalent in the tropical regions. However, dengue status is currently being reported in countries that fall in semi-arid or subtropical areas or non-endemic regions, such as Australia, the United States, and Cuba. Also, the dengue situation is worse in countries with low GDP countries, such as Malaysia, Vietnam, and the Philippines. The reason for this worse condition is due to inadequate health facilities and low GDP in these countries [70]. The relation of dengue cases with rain has been reported, but it has been discussed in this paper that apart from rain, human behavior, and social distance also affect dengue cases. The following section combines the correlation of seasonal patterns and effects on dengue in few areas across the globe based on their geographical location, socioeconomic aspects, and weather conditions.

**Table 2:** Dengue situation in the under-developed world based on GDP, climatic conditions, and rainfall [PAHO/WHO (Pan American Health Organization) 2021 [20, 71], Center for disease control and prevention 2021, Worldometer- GDP, 2021] [72]

Country	GDP (USD)	Contribution to world GDP	Dengue condition	Climatic condition	Average yearly rainfall(mm)	Level of risk
Nicaragua	$13.81 billion	0.02%	Endemic	Tropical	2,280	Frequent/Continuous
Pakistan	$305 billion	0.38%	Endemic	Semi-Arid	494	Frequent/Continuous
Cambodia	22.16 Billion	0.03%	Endemic	Tropical	1904	Frequent/Continuous

**Table 3:** Dengue situation in the developing world based on GDP, climatic conditions, and rainfall [PAHO/WHO (Pan American Health Organization) 2021 [20, 71], Center for disease control and prevention 2021, Worldometer- GDP, 2021] [72]

Country	GDP (USD)	Contribution to world GDP	Dengue condition	Climatic condition	Average yearly rainfall(mm)	Level of risk
India	2.651 Trillion	3.28%	Endemic	Tropical and subtropical	1083	Frequent/Continuous
Malaysia	315 Billion	0.39%	Endemic	Tropical	2875	Frequent/Continuous
Philippines	314 Billion	0.39%	Endemic	Tropical	2348	Frequent/Continuous
Colombia	314 Billion	0.39%	Endemic	Tropical	3240	Frequent/Continuous
Vietnam	224 Billion	0.28%	Endemic	Tropical	1821	Frequent/Continuous
Cuba	96.85 Billion	0.12%	Non-endemic	Subtropical	1335	Frequent/Continuous
Sri Lanka	87.36 Billion	0.11%	Endemic	Tropical	1712	Frequent/Continuous
Panama	62.28 Billion	0.08%	Endemic	Tropical	2928	Frequent/Continuous
Nepal	24.88 Billion	0.03%	Endemic	Subtropical	1500	Frequent/Continuous
Honduras	22.98 Billion	0.03%	Endemic	Tropical	1976	Frequent/Continuous
Bangladesh	$250 billion	0.31%	Endemic	Semi-Arid	2,666	Frequent/Continuous

**Table 4:** Dengue situation in the developed world based on GDP, climatic conditions, and rainfall [PAHO/WHO (Pan American Health Organization) 2021 [20, 71], Center for disease control and prevention 2021, Worldometer- GDP, 2021] [72]

Country	GDP (USD)	Contribution to world GDP	Dengue condition	Climatic condition	Average yearly rainfall(mm)	Level of risk
United State	19.485 Trillion	24.08%	Non-endemic	Semi-arid &Subtropical	715	Sporadic/Uncertain
Australia	1.323 Trillion	1.64%	Non-endemic	Semi-arid	534	Frequent/Continuous

#### Dengue in underdeveloped and developing nations

Dengue fever is quite dangerous and fatal at prehistoric times. It affects nearly 50 million to 100 million individuals, including the lives of children around the world in a year. Wijayanti et al. 2016 [73] has proved that socioeconomic variables also impact the dengue burden along with environmental variables, such as temperature and rainfall. It is quite prevalent in the underdeveloped and developing countries in the world due to a poor health care system. In the developing world, young women, mothers, children, and other vulnerable adults are more prone to be infected with the dengue virus. Previous research has focused on the impact of dengue burden on the gross national product of nations [74]. Not only the dengue health care is impacted due to the economic marginality, but the disease substantially reduces the output from the employable workforce in developing as well as developed nations. Montibeler et al. (2018) [74] have argued that in 2013, Brazil incurred a total loss of 0.02% of GDP due to this disease.

#### Dengue in developed nations

One of the very common causes which have been noticed in developed nations, around 30 to 100 cases of dengue are reported every year in individuals who have traveled to tropical countries. However, it was reflected that such type of cases was not even recorded. *A. aegypti* and *Aedes albopictus* are efficient vectors in developed nations. Because of these two vectors, there is a high risk of secondary spread of dengue. The spread of dengue outbreaks in the southern United States is still a potential threat, specifically in places of Gulf of Mexico. As it is quite known that from the requirement for sequential infections, DHF has been rare in travelers and does not currently pose a threat to the developed world [43].

In order to study the seasonal trends that are actively involved in dengue cases, a comprehensive data report is prepared which comprises altered dengue cases in relation to rainfall in the world. The collected data is systematically driven from Australia, Sri Lanka, India, and many other countries to establish a graphical representation-based result analysis. The graphs presented later in the section will help to determine the pattern of dengue occurrence and to find whether the rise in dengue cases is rainfall-dependent or not.

#### Rainfall/ precipitation in correlation with dengue cases

Weather is believed to be in correlation with many diseases, but vector-transmitted disease dengue does not show any pattern in relation to rainfall. It is evidently observed while trying to draw a relation. Thus, the relationship between rainfall occurrence and dengue incidences reported from 2015 to 2019 in countries from each group.

##### Nicaragua

**Fig. 3:**
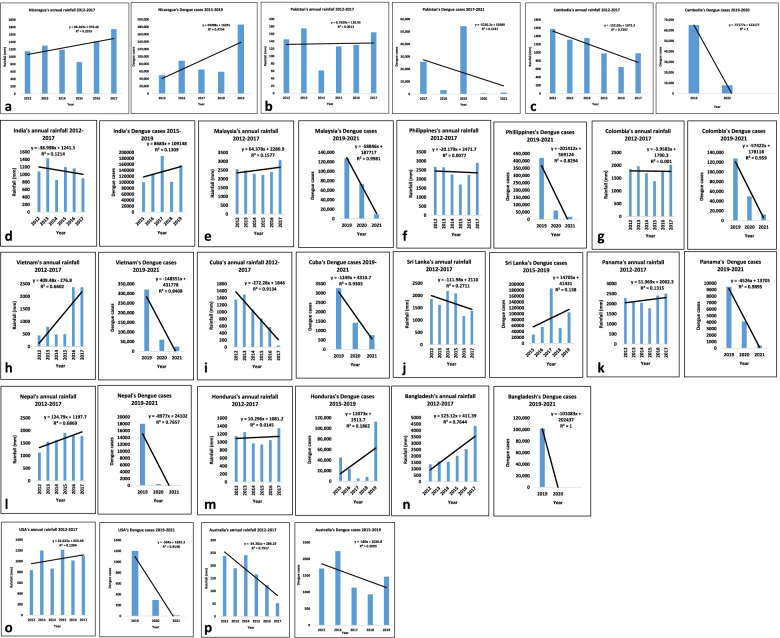
(**a**-**p**) Graphical representation of annual rainfall of different countries from 2015 to 2019 in comparison to annual dengue cases (considering the fact of availability of the data). (**a**-**c**),(**d**-**n**) and (**o**-**p**) represent the annual rainfall/dengue cases from the under-developed, developing and developed nations, respectively. Rainfall data of 2018–20 are not available at reliable sources, hence excluded from the graphs. (Source: NASA power Data access viewer [75] and European Centre for Diseases Prevention and Control [76])

In the case of Nicaragua, which lies on the American continent, it has been found that there is no correlation between rainfall patterns and the number of dengue cases (Fig. 3a). The documented rain data was found to be 855.18 mm, 1420.31 mm, and 1750.08 mm in the years 2015, 2016, and 2017, respectively. The highest number of cases of dengue (1,86,173) was reported in the year 2019 with a rainfall of 3.05 mm.

##### Pakistan

As depicted in Fig. 3b, the rainfall data is available from 2012 to 2017 whereas the dengue data is available in the literature from 2017 to 2021. In order to compare and draw some scientifically conclusive remark, we will be focusing on the relationship between the rainfall pattern and dengue cases for the period of 2017 to 2019 (Fig. 3b). The number of dengue cases was 25,872 with respect to the rainfall of 164.09 mm in the year 2017, 3204 cases with respect to 0.33 mm of rainfall in the year 2018 and 54,386 cases with respect to 0.96 mm of rainfall, and this proves that there is no strict correlation between two domains.

##### Cambodia

As Cambodia falls in the same category of underdeveloped nations with respect to Nicaragua, but the data was not sufficient to draw any direct correlation between the rainfall pattern and dengue cases. The rainfall data was only available during the period of the 2012 to 2017 year, whereas dengue cases were available from 2019 to 2021, so in this case, any definite conclusion cannot be drawn (Fig. 3c).

##### India

In the list of the developing nation, India is an example that represents a whole range of data from 2012 to 2017. The rainfall data were available for the years 2015 to 2017 and found to be 1198.08 mm, 1162.47 mm, and 901.37 mm, respectively. The dengue cases were reported as 99,913, 129,166, 188,401, 101,192, and 157,315, respectively, for the years of 2015 to 2017 (Fig. 3d). This shows that in a developing nation like India, there is an insignificant correlation between the rainfall pattern and the number of dengue cases during the last 5 years.

##### Malaysia

The countries like Malaysia where the weather is quite unpredictable because of their geographical location, the dengue cases were shown to be reduced from more than 1,20,000 (in the year 2019) to less than 80,000 (in the year 2020), which was further reduced to less than 20,000 in the year 2021. This shows the impact of the first and second wave of COVID-19 on the number of dengue cases (Fig. 3e).

##### Philippines

The Philippines is a country that is comprised of several islands. If we compare, we observed that the number of dengue cases in the year 2019 was more than 4,00,000 which further reduced to 50,000 cases in 2020 and this signifies the impact of COVID-19 on dengue cases. The impact of COVID-19 s wave was even larger as the cases were more reduced to less than half of as compared to the year 2020 (Fig. 3f).

The same pattern went with other developing nations, such as Colombia, Vietnam, Cuba, Panama, Nepal, and Bangladesh (Fig. 3g-i, k, l, and n) where the number of cases got reduced by 45% to 90%.

##### Sri Lanka

Sri Lanka is a country that is surrounded by water bodies. The annual rainfall of Sri Lanka during the duration of 2015 to 2017 was 2091.23 mm, 1173.37 mm, and 1368.49 mm, respectively. If we compare the rainfall pattern with dengue cases, we observed that there is no definite relationship is being followed. For instance, with 2091.23 mm rainfall of the year 2015, the number of dengue cases was 29,777 (Fig. 3j). The dengue cases were increased to 55,150 in the year 2016 and decreased to 186,101 in the following year of 2017. In the year 2018, where the rainfall was 4.2 mm, the dengue reported cases were found to be 51,659 and the very next year in 2019, the reported number of dengue cases were 10,5049, where the rainfall was approximately 4.68 mm. Thus, there is no definite relationship was observed between the rainfall pattern and the number of dengue cases in the case of Sri Lanka.

##### Honduras

Honduras is situated in the American continent. The rainfall during the period of 2015 to 2017 was 942.44 mm, 1051.25 mm, and 1345.32 mm, respectively (Fig. 3m). The reported number of dengue cases for the same duration of 2015 to 2019 was 44,834, 22,961, 5217, 7942, and 1,12,708, respectively. It has been observed that there is no definite relationship between the rainfall pattern and the number of dengue cases during the last 5 years.

##### Australia

Australia lies in the southern hemisphere. The rainfall pattern during the period of 2015 to 2017 was approximately 165.5 mm, 123.3 mm, and 52.36 mm, respectively. The number of dengue cases reported during this duration 2015 to 2019 were 1713, 2238, 1135, 932, and 1466, respectively (Fig. 3p).

In the underdeveloped category of Nicaragua and Pakistan, the R^2^ values (0.0013–0.4754) for the correlation between Dengue and Rainfall are less than 0.5 whereas in the case of Cambodia, the R^2^ values for the correlation between Dengue and Rainfall are 0.7297 and 1, respectively and this signifies that variable correlation between the two factors in the same category for different countries.

Similarly, for developing countries categories, the R^2^ values for India, Malaysia, Philippines, Colombia, Sri Lanka, Panama and Honduras are less than 0.5 (0.001–0.4754) except Vietnam, Cuba and Nepal, which R^2^ values (0.6602–1) are more than 0.5 between the two parameters of rainfall and dengue and this signify that they have a variable correlation between two factors in the different countries of the developing nation.

Furthermore, for developed nations, the R^2^ values for the United States of America and Australia are less than 0.5 (0.1394–0.3095) and this found that the two factors are weakly correlated with each other Fig. 3 (o-p).

Based on R^2^ values of rainfall and dengue of different categories, it has been deciphered that there is no direct correlation that can be ascertained between two factors when they are considered globally. The fact that dengue mosquitoes breed in clean water unlike other vector-borne diseases such as malaria. The source of clean water can be rainfall as well as clean water in domestic spaces of the urban localities. Hence, rainfall cannot be the only determinant factor for the spread of dengue.

**Fig. 4:**
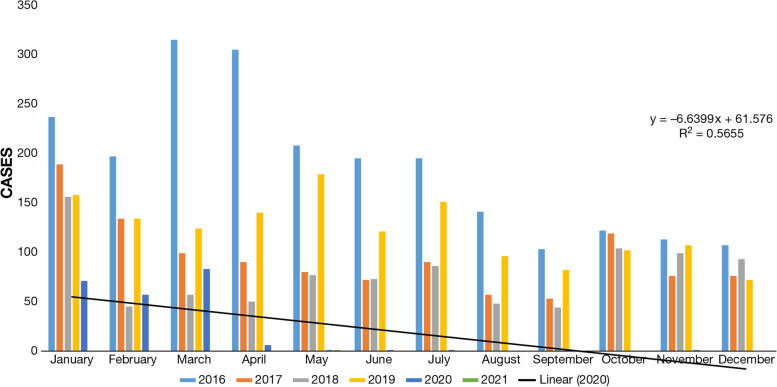
Dengue cases month-wise from 2018 to 2021 in Australia. Source: Prevention, National notifiable diseases surveillance system (Australia Government), 2021 [77]

Australia is a curious case as it falls in the category of a developed nation and has a major arid zone. We compared dengue cases from 2018 to 2021 around the year. The number of cases reported in the year 2020 and 2021 are far less than any year from 2018 to 2019 (Fig. 4). For instance, the number of cases in January 2020 was 71 and which got further reduced to 1 case in 2021 (Data is until May 2021). On the other hand, in the months of February and March 2020, dengue cases were reported to be 57 and 83, respectively, and following this, the number of cases for the year 2020 came down to single digit which was 6 in the month of April, and only a single case was observed in the months of May, June, July, and November. In the months of August, September, October, and December, 0 cases were reported. For the year 2021, only a single case is reported till May 2021. Meanwhile, if we compare dengue cases from the year 2018 to 2019, the cases were jumped up to three-digit. The value of *R*^2^ is 0.5655, which indicates that the correlation between the two (i.e., dengue cases and COVID-19) is quite high.

In the recent years, Sri Lanka has become a center of vector-borne diseases, such as malaria and dengue. In the South Asian region, it is one of the worst-affected countries with dengue fever.

**Fig. 5:**
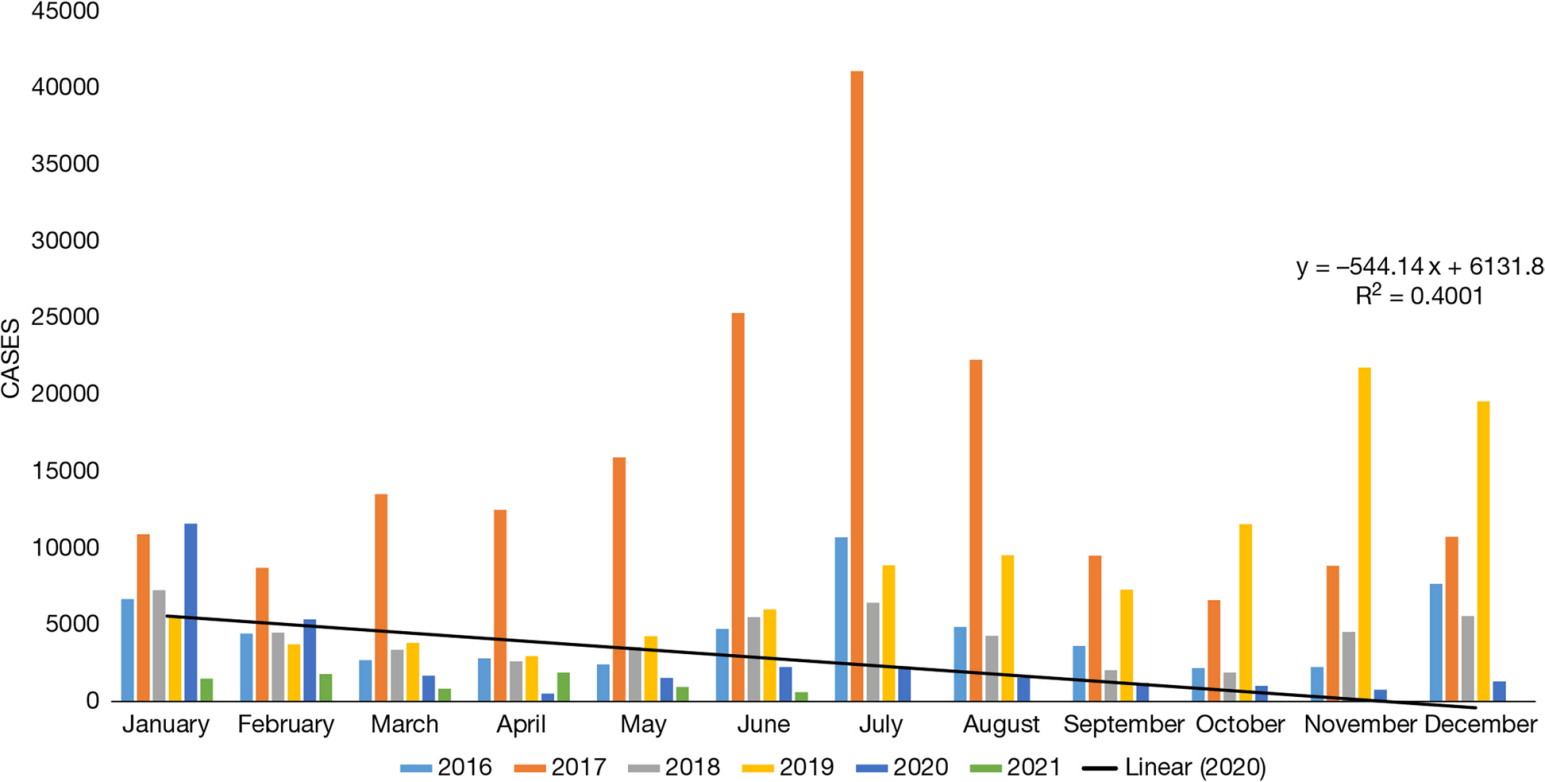
Dengue cases month-wise from 2018 to 2021 in Sri Lanka. Source: Epidemiology Unit Ministry of Health (Sri Lanka Government), 2021 [78]

The number of dengue observed in the years 2020 and 2021 are reported less in number than the years of 2018 to 2019. Except for January 2020, where the reported number of dengue cases was 11,608, in every month of the year 2020, the cases were comparatively low. In the month of February, March, and April, the cases were 5368, 1683, and 511, respectively. In the month of November 2020, the number of cases came down to three digits as 770. As of May 2021, the number of cases is reported in the year 2021 is significantly low in comparison to all other years. Except April 2021, in all the months of the year, the number of dengue cases were reported lowest in number as compared to other years. For instance, in January to March 2021, the number of dengue cases were 1496, 1794, and 849, respectively, whereas, in the month of May and June 2021, the number of cases was 959 and 617, respectively (Fig. 5).

In most parts of Australia, the monsoon extends from December to March, so the number of dengue cases is also recorded in these months [20]. Similarly, the monsoon in Sri Lanka occurs between May to September, causing dengue cases to become more severe in July and August [20]. When a linear line is drawn in Australia for April, its *R*^2^ value is 0.5655, which means dengue cases follow the trend of large-scale rainfall.

##### Seasonal trend of dengue in India

The weather of India can be divided into three parts: pre-monsoon, monsoon, and post-monsoon. Pre-monsoon occurs from February to May, monsoon occurs from June to mid-September, and post-monsoon occurs from mid-September to January [41]. Dengue cases are seen more in the post-monsoon period than in the pre-monsoon and monsoon [79]. This is because the dengue mosquito larva cannot survive due to low temperature and low rainfall during the pre-monsoon period. The pre-monsoon period is from late January to May. In February, the night temperature is below 10 °C in many states of India and low rainfall during the following month which causes unfavorable conditions for the dengue’s larvae to survive. The appropriate temperature for dengue larvae to grow is between 15 °C and 35 °C [80]. Moreover, the larva cannot survive due to excess rainfall during the monsoon period. In the post-monsoon periods, the rain stops, the water accumulates in the household waste and open places near the houses. All these conditions including the average temperature are more suitable for the breeding of dengue mosquitoes. At these times, more cases of dengue are recorded in different regions of India. The number of dengue cases is highest between mid-September to the end of October [81]. The graphs discussed later in the section will demonstrate segregated data analysis of seasonal patterns and dengue in different parts of India, such as Rajasthan, Madhya Pradesh, Gujarat, and many other states which come under semi-arid regions and show different seasonal pattern than other states of India.

### Negative impact of COVID-19 on dengue fever

#### Impact of COVID-19 lockdown and social distance on dengue cases

Worldwide lockdown to prevent infection of COVID-19 has also affected the prevalence of many other infectious diseases, of which dengue fever is also a significant disease [4]. The breeding site of mosquitoes responsible for dengue fever is more prevalent indoor and outdoor environments [27]. It has been observed that the behavioral changes in the restriction travelling of people due to lockdown influences a lot in the number of dengue cases in major parts of the countries [82]. People were in lockdown at home for 3 to 4 months [26, 27], due to which they were not in contact or infected with mosquitoes in the outside environment (Figs. 7, 8 and 9). It has been seen that the number of dengue cases recorded before the lockdown was almost the same as recorded with the last year (before the year 2019). These inferences draw a major impact of lockdown on the number of cases of dengue during the period of 2020. The limited exposure to the outdoor environment significantly flattened the dengue curve when compared with the previous year’s data.

**Fig. 6:**
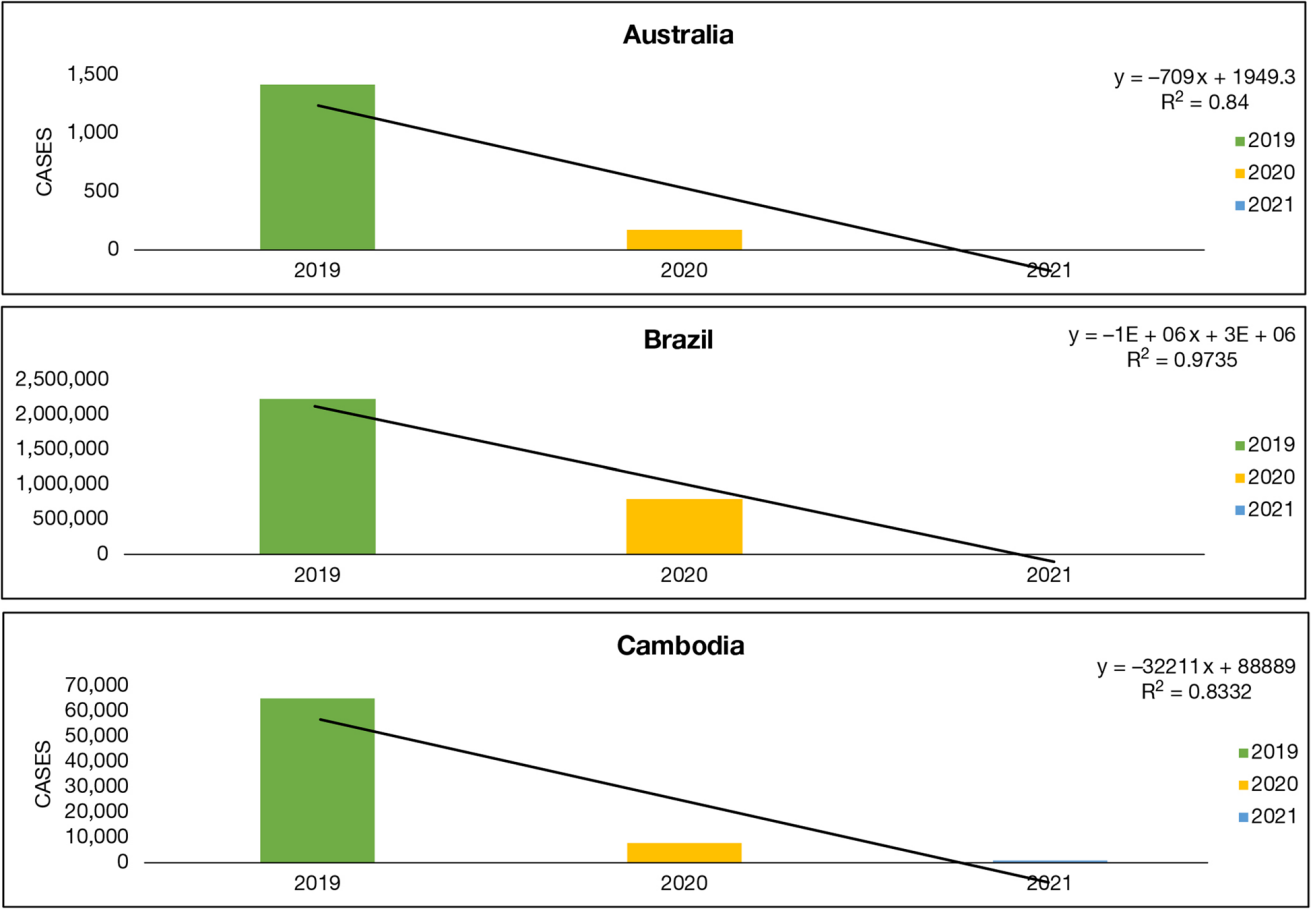
Dengue cases in arid region countries: Australia, Brazil, and Cambodia between 2019 and 2021. Source: According to CDC (Center for diseases control [83], PAHO (Pan America health organization [20])

Countries which fall in a geographically arid region, such as Australia, Brazil, and Cambodia, which includes a mixed group of the under-developed and developed nation simultaneously have some interesting facts to study (Fig. 6). Australia in the year 2019 reported 1419 dengue cases which came down to 174 in the year 2020 and further reduced to a 1 by May 2021. Brazil, which is GDP-wise average, recorded 22,25,461 dengue cases in the year 2019; however, the cases then drastically dropped to 7,94,565 in the year 2020 and 2,29,446 until May 2021. Cambodia is a poverty-stricken country and these vector-borne diseases, such as dengue and malaria are more prevalent there. It had also been reported a similar pattern as 65,000 cases of dengue was reported in the year 2019 and 7823 cases in the year 2020, consequently 578 until May 2021. The *R*^2^ value is 0.84,0.9735 and 0.8332, respectively, for Australia, Brazil, and Cambodia, which indicates the fact the relationship between these two ailments is noteworthy.

**Fig. 7:**
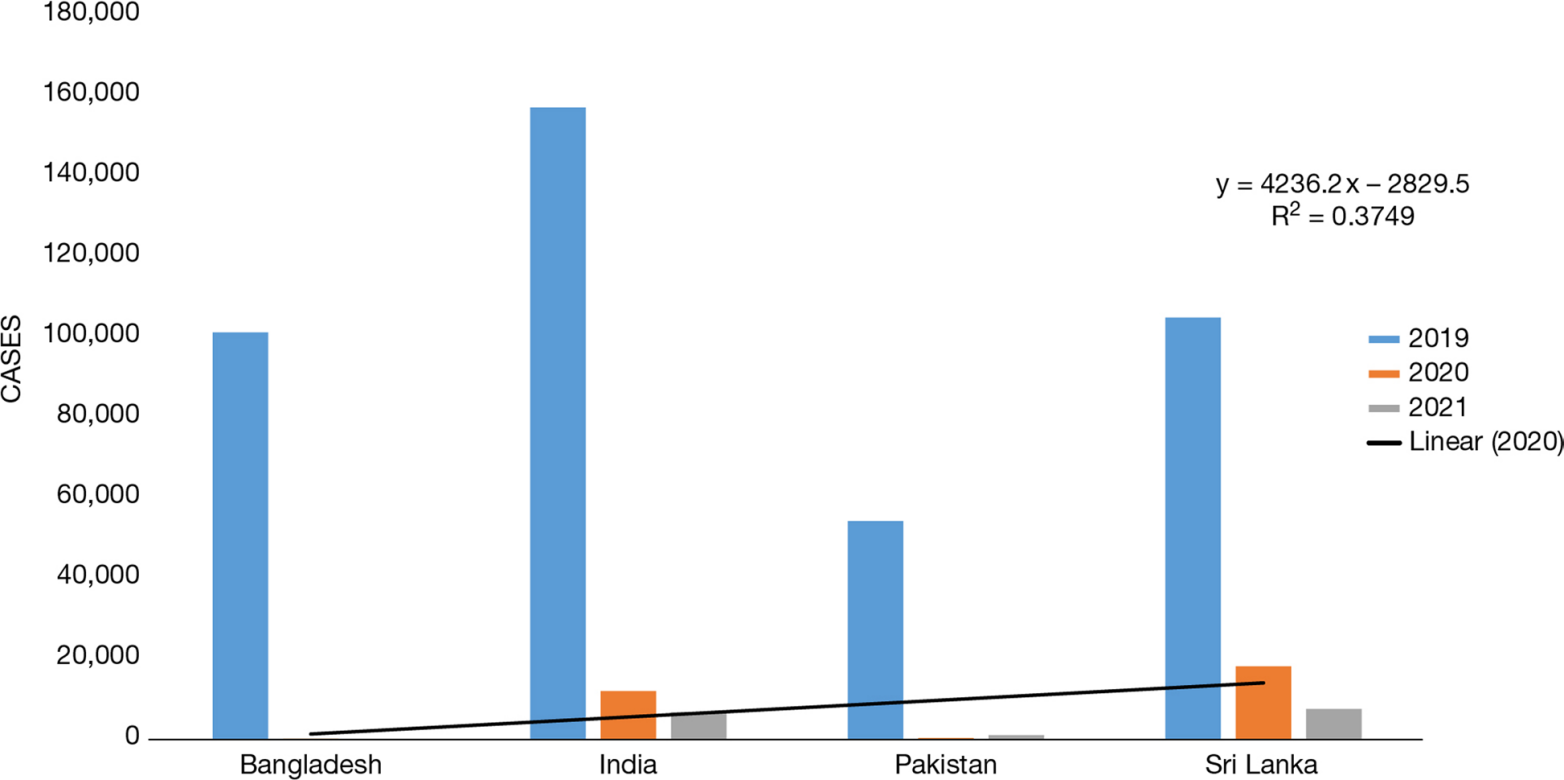
Dengue cases in semi-arid countries: Bangladesh, India, Pakistan and Sri Lanka during 2019 to 2021. Source: According to CDC (Center for diseases control) [83], PAHO (Pan America health organization [20])

The semi-arid landscape nations such as Bangladesh, India, Pakistan, and Sri Lanka are considered to be underdeveloped and developing nations which signify the fact that their health care system is not well developed (Fig. 7). When compared to cases reported in Bangladesh in the year 2019, the number of dengue cases was 1,01,354 which remarkably came down to 271 in the year 2020 and for 2021 they have not been reported. In populated countries like India and Pakistan, the number of cases in the year 2019 was 1,57,315 and 54,386, respectively, which were notably reduced to 12,078 and 416, respectively, in the year 2020. For the year 2021 until May, the number of cases for India, Pakistan, and Sri Lanka was 6837, 1115 and 7616, respectively, which was significantly less than other years.

**Fig. 8:**
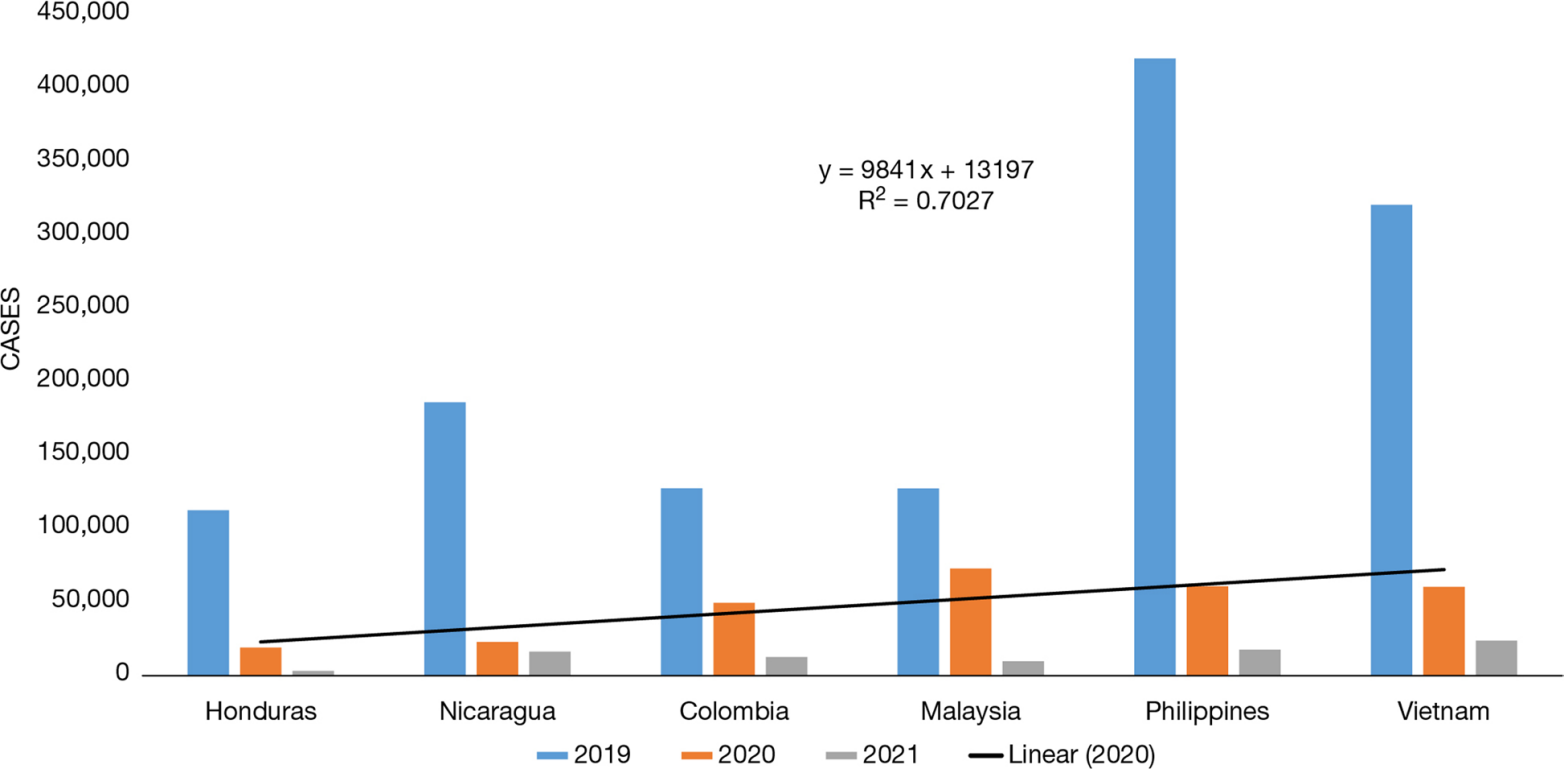
Dengue cases in tropical countries: Honduras, Nicaragua, Columbia, Malaysia, Philippines, and Vietnam from 2019 to 2021. Source: According to CDC (Center for diseases control) [83], PAHO (Pan America health organization [20])

It is a well-postulated fact that the tropical region is best suited for the dengue vector to breed. In the tropical region, the number of vector-borne diseases is always high in comparison with semi-arid or arid regions because of the availability of water and temperature which is appropriate for the vector to breed. Some countries are chosen in order to study the trend in the tropical territory. The number of cases reported in the year 2019 in Honduras, Nicaragua, and Colombia was 1,12,708, 1,86,173, and 1,27,553, respectively, but in the year 2020, the number of cases was reduced to 19,037, 22,951, and 49,561 for the same, respectively (Fig. 8). Countries, such as Malaysia, Philippines, and Vietnam were turned up with 1,27,407, 4,20,453, and 3,20,702, respectively, with dengue cases in the year 2019 which got significantly reduced to 72,952, 60,819, and 60,525, consequently in the next year 2020. The year 2021 reported the lowest number of dengue cases as of May 2021. For instance, Honduras, Nicaragua, Colombia, Malaysia, Philippines, and Vietnam reported 3343, 16,465, 12,710, 9715, 17,630, and 24,000, respectively, in the year 2021.

These graphs (Figs. 6, 7 and 8) compare the dengue cases of the affected countries of the world during the years 2019 to 2021. According to Figs. 6, 7 and 8, it may be inferred that all these countries have fewer cases reported in 2020 and 2021 than in 2019. Another reason which is noteworthy is cross-reactivity and overlap of protective immunity which has its drawback as well but at the same time, it helps in the development of antibodies that fights with the vice versa ailment.

**Fig. 9:**
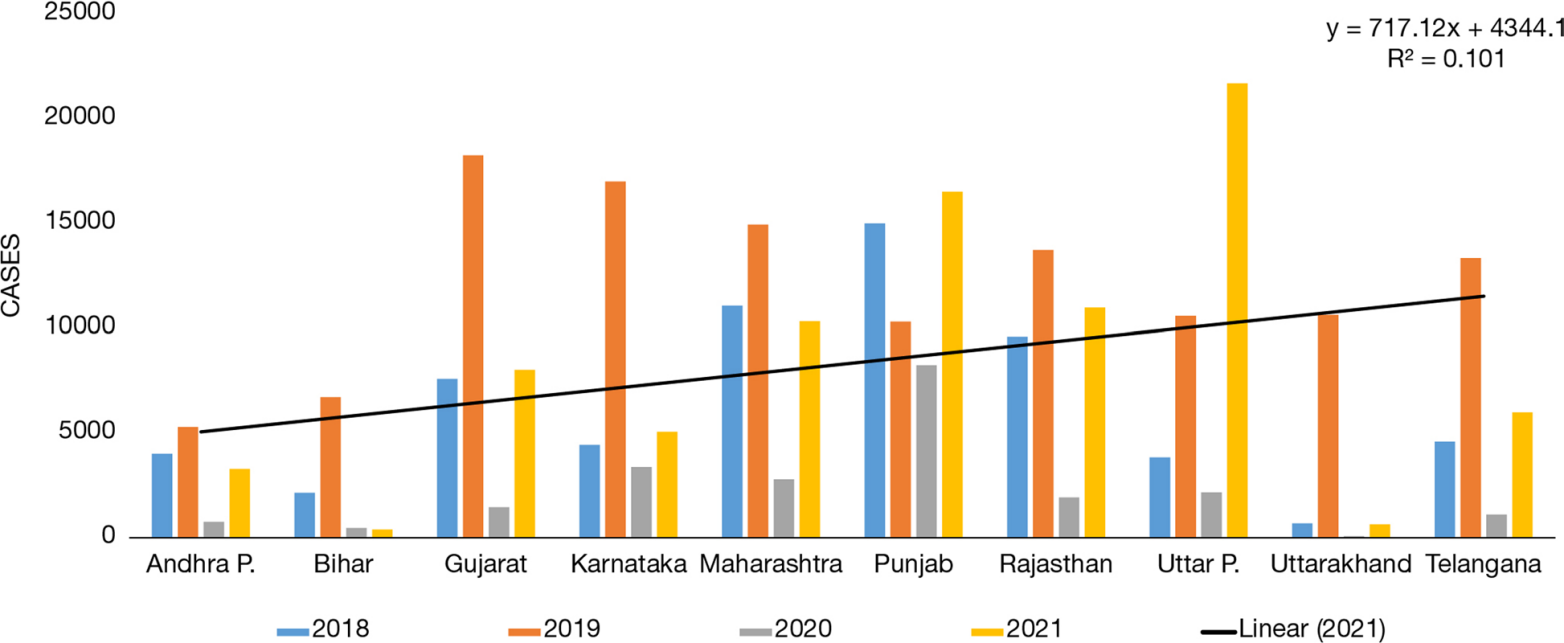
Dengue cases situation in Indian states between 2018 and 2021. Source: NVBDCP, [38] (National Vector Borne Disease Control Programme) Govt. of India

India is a country with a large landmass which comprises of arid (i.e., Punjab, Karnataka, Gujarat, and Rajasthan); semi-arid (Maharashtra, Andhra Pradesh, Bihar, Uttar Pradesh, and Uttarakhand); and tropical regions (Telangana) and if we compare the dengue cases of its internal states, the same pattern is being observed as the number of dengue cases in the years, 2020 and 2021 are way too less in comparison to the past 2 years 2018 and 2019 (Fig. 9). The same states reported a far less number of cases, such as Bihar and Uttarakhand reporting 0 cases, whereas Punjab recorded only two-digit cases, that is, 37. However, Andhra Pradesh, Gujarat, Karnataka, Maharashtra, Rajasthan, Uttar Pradesh, and Telangana reported 461, 201, 903, 530, 826, 105, and 237 cases, respectively.

**Fig. 10:**
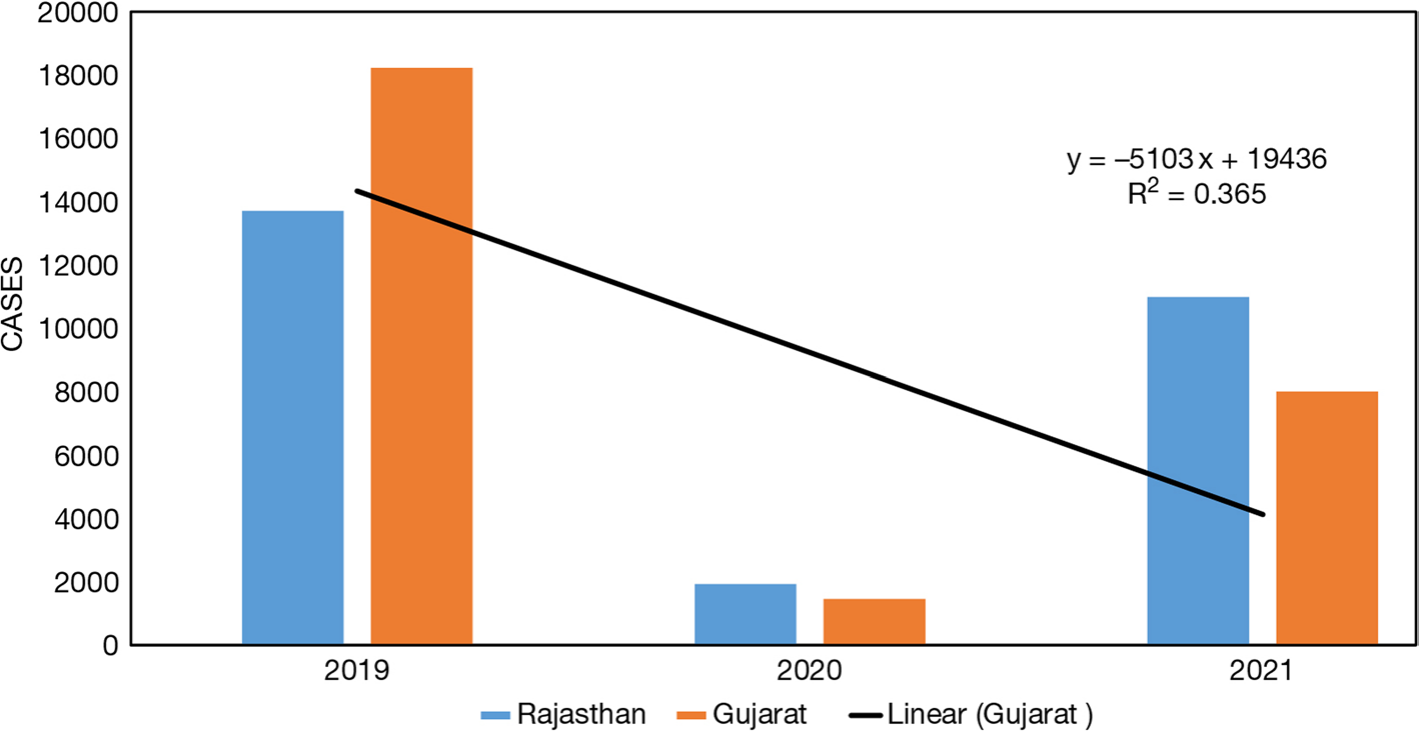
Dengue cases in arid states of India: Rajasthan and Gujarat from 2019 to 2021. Source: NVBDCP, [38] (National Vector Borne Disease Control Programme) Govt. of India

Basically, an arid climate is characterized as one in which for a major part of the year, precipitation is less than the potential evapotranspiration. The rainfall in this region is usually low and the distribution is unequal. The northern arid regions in India comprise largely of the desert of Rajasthan and Gujarat (Fig. 10). We compared the dengue cases in states, such as Rajasthan and Gujarat before and during the pandemic year 2019 to 2021. In the year 2019, the number of dengue cases reported in Rajasthan and Gujarat, respectively, was 13,706 and 18,219, respectively, which significantly reduced in Rajasthan to 1929, and cases in Gujarat dropped to 1458 in the year 2020. The year 2021 reported 10,984 and 8013 cases, respectively, for the states of Rajasthan and Gujarat. The *R*^2^ value is 0.365, which is quite valuable to consider, extrapolates the relation between the two diseases.

**Fig. 11:**
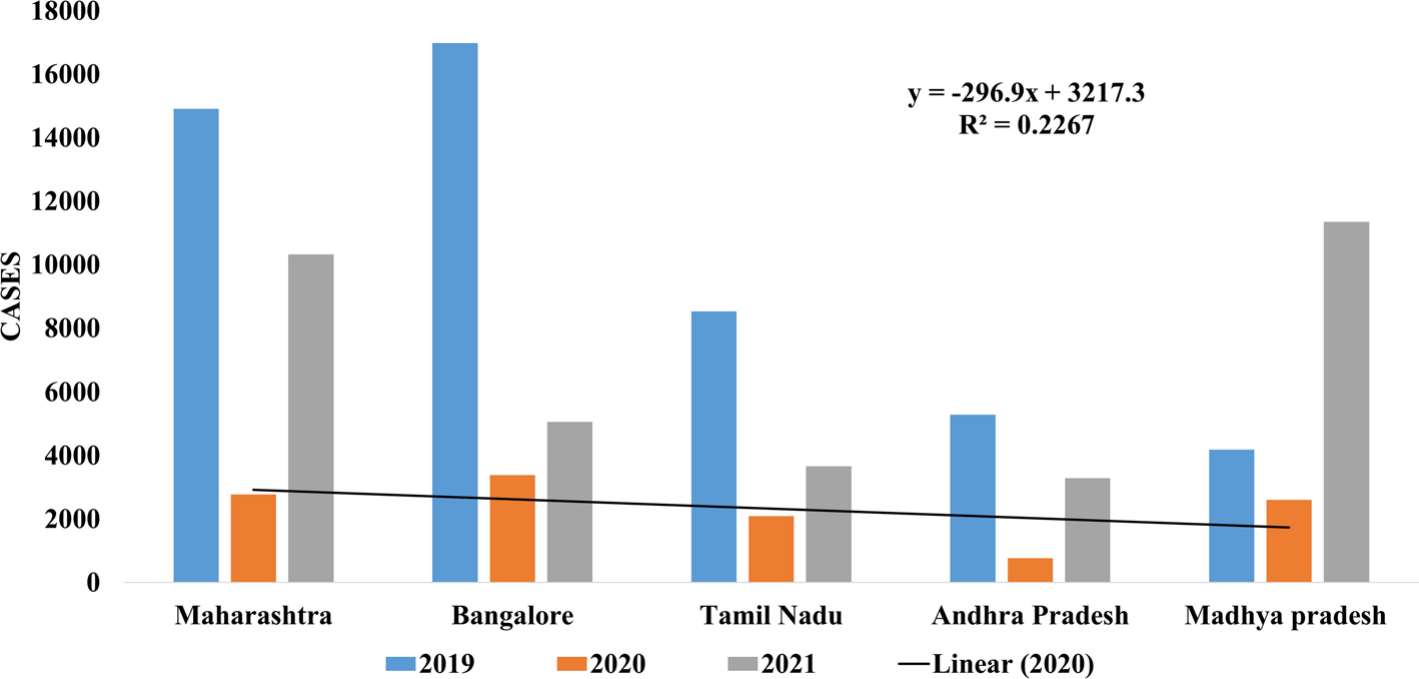
Dengue cases in semi-arid states of India: Maharashtra, Bangalore, Tamil Nadu, Andhra Pradesh, and Madhya Pradesh between 2019 and 2021. Source: NVBDCP, [38] (National Vector Borne Disease Control Programme) Govt. of India

Indian states, such as Maharashtra, Bangalore, Tamil Nadu, Andhra Pradesh, and Madhya Pradesh are majorly situated in semi-arid zone (Fig. 11). In states like Maharashtra, Karnataka, and Tamil Nadu, dengue cases reported in the year 2019 were 14,907, 16,986, and 8527, respectively; but, during the pandemic year 2020, at the same time zone, the number of cases reduced to 2781, 3384, and 2096, respectively. The year 2021 reported a lower number of cases comparatively as for Maharashtra (10,320), Karnataka (5062), Tamil Nadu (3665), Andhra Pradesh (3285), and Madhya Pradesh (11,354) cases were there.

**Fig. 12:**
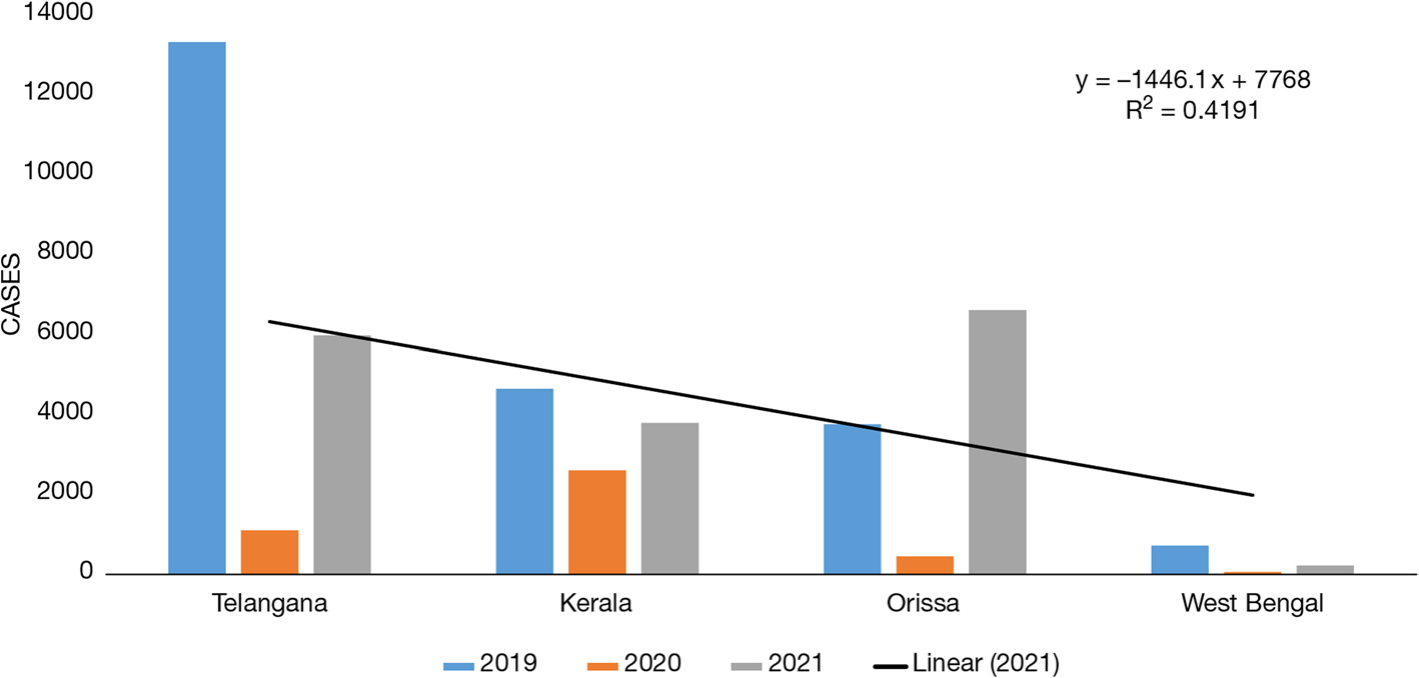
Dengue cases in tropical states of India: Telangana, Kerala, Orissa, and West Bengal from 2019 to 2021. Source: NVBDCP, [38] (National Vector Borne Disease Control Programme) Govt. of India

When it comes to tropical states and their comparison between the years 2019 and 2020, then the results become even more important as it is an established fact that tropical regions are suitable environments for the breeding of dengue mosquitoes. The states, such as Telangana, Kerala, Orissa, and West Bengal are included in the tropical region, and the number of cases observed in the year 2020 was quite less in comparison to the year 2019 (Fig. 12). If we observe the states Telangana, Kerala, and Orissa, the number of cases decreased from 13,331, 4632, and 3758, respectively, in the year 2019 to 1108, 2605, and 455, respectively, in the year 2020. States like Telangana, Kerala, and Orissa reported 5983, 3794 and 6610 number of cases of dengue in the year 2021.

#### Potential serological cross-reactivity in dengue and COVID-19 cases

Tropical areas are pressurized with the rapid increase of COVID-19 cases in tropical as these areas are earlier facing health challenges every year due to dengue fever. In some of the cases of COVID-19, the presence of symptoms similar to dengue has become a problem, as both diseases have similar clinical and laboratory features. The highest impact will be on low-income countries in the tropical region, because their health services are not as advanced as other countries [63, 84–86].

Similarly, in the research conducted in Israel, out of 55 COVID-19 cases, 12 cases of rapid test turned positive for dengue with IgM and IgG antibodies. However, after 1 week, this test proved negative in these patients [84, 86]. In another case, a 14-year old patient complained of an initial fever, headache, after which his fever increased to 103 °C. After few days, the patient also felt nausea, vomiting, and after the clinical diagnosis of this patient, it was found a platelet count of 70 × 10^3^ per mm^3^, a leukocyte count of 2620 per mm^3^ that was much lower than average. The C-reactive protein levels, D-dimer, and serum ferritin were high in this patient, reflecting the COVID-19 status. Simultaneously, a rapid serological test for dengue fever came positive for NS1 antigen and IgM, which confirmed dengue with COVID-19 [84]. Thus, the presence of two virus-borne diseases in the same patient at the same time may prove to be a global challenge. All these cases indicate cross-reactivity or overlapping between dengue and COVID-19, which may have a massive impact on health services.

## Discussion

The common antigenic cross-reactivity and the life cycle of dengue and COVID-19 virus is quite similar as they both bind to the host surface receptors leading to its internalization through endocytosis, after entering the virus releases its RNA which is further uncoated. RNA copies are made and after packaging the viruses are released through the exocytosis process. Antigenic cross-reactivity between dengue and COVID-19 augment few other perturbations as well. One of them is the possibility of protective immunity overlap umbrella but, on the other side, it can aggravate the situation as well by increasing the amount of antibodies production in the body, which is not required otherwise. The basic similarity between the SARS-CoV-2 and dengue is partially sufficient in explaining the cross-reactivity nature of these two. Another issue with having similarity between these two is that at times it gives false-positive tests for COVID-19 and vice and versa (Fig. 1). Thus, misdiagnosis of both the diseases leads to poor prognosis and interferes with other urgent necessary management for the prevention of infections. In addition, Zika virus outbreaks have been reported in the states of Kerala and Maharashtra of India in the month of July 2021 and its infection is similar to COVID-19 which might lead to misdiagnosis and thus further lead to underreporting of cases [87]. In 2019, a total of 10,768 plausible cases of Zika infection were reported in Brazil, followed by the first cases of COVID‐19 in February 2020 with the outbreak of 10 million COVID‐19 cases in early March 2021. The epidemic of Zika infection had increased in Brazil during the year of 2015 because of a lack of access to water, hygiene, and unequal access to health care for the most impoverished families [88–90].

Vaccination also poses a challenge in controlling the COVID-19 cases. According to the Indian database, approximately 7.03% of the total population has been fully vaccinated against COVID-19 with the first and second dose in late July 2021 [91, 92]. In other ASEAN countries, Cambodia and Malaysia have the second-highest rate of inoculation at 47%, followed by 11% and 7% in the Philippines and Vietnam of vaccination rates, respectively of the entire population [34, 93]. Due to the shortage of vaccination, the under-health care system and other influencing factors such as misdiagnosis, false-positive results hinder the control of dengue and COVID-19 [94].

The healthcare systems of the under-developed countries such as India and Pakistan are facing major setbacks due to a lack of necessary preventive measures to fight this outbreak, and healthcare workers (HCWs) are also reaping the consequences. During the second wave, a sudden rise in COVID-19 cases in India, with a record-breaking > 400,000 cases daily, was caused by the emergence of another variant such as double mutant or B.1.617 variant. Only 8.5 hospital beds and 8 physicians were available per 10,000 population [95–97] whereas in Pakistan, only 0.6 beds/1000 people are available during the pandemic. In addition, in the case of Pakistan, less than 0.75 percent of GDP is used for the healthcare system. Thus, it was the worse conditions for the underdeveloped countries to tackle the outbreak of COVID-19 [32, 98].

This article explores various factors responsible for the incidence of dengue and COVID-19. The association of COVID-19 including waves 1 and 2 with its consequences which arose from lockdown and dengue cases in the year 2019 to 2021 are studied carefully with collected data from different parts of the world. The data was classified on many factors like geographical zones for instance arid, semi-arid, and tropical regions; besides this another categorization was on underdeveloped, developing, and developed nations. These parameters, such as geographical locations and a nation’s GDP status have acted as a control to our article in order to reach a conclusive remark (Tables 2, 3 and 4). When we compared the data of dengue cases before and during this pandemic across the globe of different regions like tropical and subtropical (arid and semi-arid), it could be concluded that there might be some correlation between a reduced number of dengue cases and behavioral changes that have been shown in society due to the after effect of lockdown. Likewise, a similar scenario comes into the picture for Indian states as well. Especially the Indian states, which are located in different zones, that is, arid, semi-arid, and tropical regions also showed a similar pattern. Uniformly all states reported a lower number of dengue cases during the pandemic year 2020 and 2021 in comparison to cases reported in previous years. The number of dengue cases tremendously declined during the COVID-19 outbreak, precisely in both the waves, which might be due to lockdown and limited human interaction in the society that restricted human to be exposed to outdoor environment and reduced the propensity of dengue disease. The major finding of the present study is that dengue is variably correlation with rainfall in different countries of the geographical locations, but it has been found that the dengue cases is inversely correlated with COVID-19, and this infer that the infectivity of the virus is less prevalent for transmission due to limited travel activities and worldwide lockdown scenarios.

The second wave of COVID-19 was quite colossal in its intensity in India itself, due to which its effect on Indian states can be seen directly in the form of reported dengue cases, which was significantly less in comparison to other previous years.

Underdeveloped, developing, and developed nations showed no correlation between the rainfalls from 2012 to 2017 with the occurrence of dengue cases. Underdeveloped nations, such as Cambodia, where dengue cases were more than 60,000 in 2019 got reduced to less than 10,000. Developing nations, such as Philippines, Colombia, and Vietnam also showed the same pattern where the cases dropped drastically from more than 4,00,000 in 2019 to less than 50,000 in 2021 [99].

If we compare the impact of COVID-19 in the second wave over the first wave, the drastic and significant reduction can be observed in dengue cases.

In the Tropical region, the COVID-19 s wave lasted for around 4 months from March to July 2021 of which the highest number of COVID-19 active cases was reported in May 2021. The number of Dengue cases reported for countries situated in the tropical geographical zones like Honduras, Nicaragua and Colombia was 3343, 16,465 and 12,710, respectively, which were low in contrast to previous years.

In India, the second wave was at its peak from April 14, 2021, to May 30, 2021 and during this period, the maximum number of active cases around 3,741,179 with COVID-19 was reported on May 9, 2021. During the same period the number of Dengue cases in different states viz., Bihar, Uttarakhand and West Bengal were reported as 96, 641 and 224, respectively.

In a similar scenario the pattern was observed in arid region countries. The number of dengue cases was lowest during the second wave in Australia (1), Brazil (2,29,446) and Cambodia (578) in comparison to Dengue cases in the previous years which were many folds than year 2021 [100]. In all categories of underdeveloped, developing, and developed countries, the number of cases got reduced by 55%, 65%, and 60%, respectively, which proves the fact that the COVID-19 s wave had some considerable impact on dengue cases. Recently, it has been reported that public movement restriction during the COVID-19 pandemic has significantly reduced the number of dengue cases in Sri Lanka [101].

This study holds novelty in terms of explaining the close association of dengue before and during an incidence of the world COVID-19 outbreak.

## Conclusions

COVID-19 and dengue fever are both viral diseases, and their status remains epidemic in the world. However, the COVID-19 cases are not yet following any seasonal pattern. The dengue cases in the year 2020 and 2021 are not following the seasonal pattern, which is due to influencing factors, such as lockdown and social distance imposed due to the COVID-19 outbreak. Human behavior has greatly changed due to lockdown and social distancing. Due to restrictions applied through lockdown, home quarantine, and social distancing, people are leaving their houses less often and avoiding gathering at crowded places. In 2020, the rainfall has been recorded equal to the previous year, but in the same year, COVID-19-imposed lockdown has changed the scenario worldwide. This shows that changes in the conduct of human beings lead to a decrease in the cases of dengue.

On the basis of data retrieved through websites, such as WHO, ECDC, and CDC, it could be categorically concluded that the trend between COVID-19 and number of dengue cases are inversely proportional to each other.

## Data Availability

The data that support the findings of this study are available from various open websites: 1. ECDC (European Centre for diseases prevention and control) [URL:https://www.ecdc.europa.eu/en/publications-data/dengue-annual-epidemiological-report-2019]. 2. Epidemiology Unit Ministry of Health (Sri Lanka Government), 2020 [URL:https://www.epid.gov.lk/web/index.php?option=com_casesanddeaths&Itemid=448&lang=en#]. 3. CDC (Center for diseases control), 2020. CDC-Dengue-Areas with risk of Dengue. [URL:https://www.cdc.gov/dengue/areaswithrisk/around-the-world.html]. 4. National Aeronautics and Space Administration (NASA) power Data access viewer, https://power.larc.nasa.gov/data-access-viewer/ 5. PAHO/WHO (Pan American Health Organization)- Dengue cases. [URL:https://www.paho.org/data/index.php/en/mnu-topics/indicadores-dengue-en/dengue-nacional-en/252-dengue-pais-ano-en.html?start=1]. 6. Prevention, National notifiable diseases surveillance system (Australia Government) [URL: http://www9.health.gov.au/cda/source/rpt_3.cfm]. 7. WHO-2019, Weekly epidemiological update on COVID-19. [URL:https://www.who.int/emergencies/diseases/novel-COVID-19virus-2019/situation-reports].
